# Analyzing the Functional Roles and Immunological Features of Chemokines in COAD

**DOI:** 10.3390/ijms25105410

**Published:** 2024-05-15

**Authors:** Houxi Xu, Yihua Song

**Affiliations:** 1 School of Chinese Medicine, Nanjing University of Chinese Medicine, Nanjing 210023, China; houxixu@126.com; 2 School of Artificial Intelligence and Information Technology, Nanjing University of Chinese Medicine, Nanjing 210023, China

**Keywords:** chemokines, COAD, prognosis, immune infiltration, prognostic risk model

## Abstract

Chemokines are key proteins that regulate cell migration and immune responses and are essential for modulating the tumor microenvironment. Despite their close association with colon cancer, the expression patterns, prognosis, immunity, and specific roles of chemokines in colon cancer are still not fully understood. In this study, we investigated the mutational features, differential expression, and immunological characteristics of chemokines in colon cancer (COAD) by analyzing the Tumor Genome Atlas (TCGA) database. We clarified the biological functions of these chemokines using Gene Ontology (GO) annotation and Kyoto Encyclopedia of Genes and Genomes (KEGG) pathway enrichment analysis. By univariate and multivariate COX regression analyses, we developed chemokine-based prognostic risk models. In addition, using Gene Set Enrichment Analysis (GSEA) and Gene Set Variant Analysis (GSVA), we analyzed the differences in immune responses and signaling pathways among different risk groups. The results showed that the mutation rate of chemokines was low in COAD, but 25 chemokines were significantly differentially expressed. These chemokines function in several immune-related biological processes and play key roles in signaling pathways including cytokine–cytokine receptor interactions, NF-kappa B, and IL-17. Prognostic risk models based on CCL22, CXCL1, CXCL8, CXCL9, and CXCL11 performed well. GSEA and GSVA analyses showed significant differences in immune responses and signaling pathways across risk groups. In conclusion, this study reveals the potential molecular mechanisms of chemokines in COAD and proposes a new prognostic risk model based on these insights.

## 1. Introduction

Colon cancer (COAD) ranks among the most prevalent malignancies worldwide. According to recent statistics, it is the second leading cause of cancer-related deaths. The incidence of this disease is associated with various factors, including genetic predisposition, aging, dietary habits, and lifestyle [1,2]. However, early-stage colon cancer often presents no significant symptoms, resulting in the majority of patients being diagnosed at advanced stages [3]. Despite the widespread use of standard treatments like surgery, radiotherapy, and chemotherapy, the 5-year survival rate for advanced colon cancer patients is only 13%, with overall survival usually under 12 months [4]. Recently, the role of biomarkers in cancer diagnosis and treatment has gained increasing recognition [5]. Several studies have been conducted to identify molecular markers linked to the onset, progression, prognosis, and treatment response of colon cancer [6]. Despite the progress made, such as the discovery of gene mutations like KRAS and NRAS and their application in targeted therapy strategies, tools for accurately predicting long-term survival and treatment responses in colon cancer patients remain limited [7]. The heterogeneity and diversity of colon cancer challenge the efficacy of relying solely on a single biomarker or simple prognostic risk models for clinical needs. Consequently, there is an urgent need to develop a new prognostic risk model incorporating multiple biomarkers.

Chemokines are a group of signaling proteins that play a central role in the immune system and inflammatory responses. They regulate leukocyte migration and overall immune functions [8], impacting various biological processes and disease states from infections to autoimmune diseases and tumor development [9]. Chemokines are classified into four main classes: CC, CXC, XC, and CX3C [10]. CC includes CCL1, CCL2, CCL3, CCL3L1, CCL4, CCL4L1, CCL5, CCL7, CCL8, CCL11, CCL13, CCL14, CCL15, CCL16, CCL17, CCL18, CCL19, CCL20, CCL21, CCL22, CCL23, CCL24, CCL25, CCL26, CCL27, and CCL28; CXC includes CXCL1, CXCL2, CXCL3, CXCL4 (PF4), CXCL4L1 (PF4V1), CXCL5, CXCL6, CXCL7 (PPBP), CXCL8, CXCL9, CXCL10, CXCL11, CXCL12, CXCL13, CXCL14, CXCL16, and CXCL17; XC includes XCL1, and XCL2; CX3C includes CX3CL1. Each subfamily has distinct structures and functions, interacting with specific receptors to activate intracellular signaling pathways. The CC subfamily attracts monocytes, eosinophils, and memory T cells, while the CXC subfamily primarily targets neutrophils [11,12]. The XC and CX3C subfamilies have cell-type-specific functions crucial for precise immune response regulation [13]. In cancer development, chemokines can have a dual role by either attracting immune cells to inhibit tumor growth or promoting tumor growth and metastasis through facilitating tumor cell migration, invasion, and angiogenesis [14]. This complex interplay makes chemokines a potential key target in cancer therapy.

Chemokines are pivotal molecules that regulate immune responses and cell migration, exerting a significant influence on the dynamic changes within the tumor microenvironment (TME). They play diverse roles in tumor development, metastasis, and immune evasion mechanisms [15]. Throughout different stages of tumor progression, chemokines attract distinct immune cell types to the TME, reshaping its composition and impacting tumor advancement and patient outcomes. Chemokines like CCL2 and CCL5 are essential for recruiting regulatory T cells, inflammatory monocytes, and macrophages, creating an immunosuppressive environment in the TME that supports tumor growth and worsens patient prognosis [16,17,18]. Consequently, targeting chemokines emerges as a novel therapeutic strategy for cancer treatment, given their role in modulating immune responses and angiogenesis within the TME [19]. For instance, in non-small-cell lung cancer (NSCLC), CXCL8 triggers endothelial cell activation via its receptors CXCR1 and CXCR2, activating the PI3K and MAPK signaling pathways to promote angiogenesis [20]. Furthermore, CXCL8 collaborates with molecules such as VEGF in the TME to collectively enhance angiogenesis, affecting tumor growth and metastasis [21]. The diversity and complexity of chemokines in tumor biology make them a significant area of research. While some understanding exists regarding the roles and mechanisms of chemokines in various tumor types, their specific functions and biological significance in colon cancer remain a key research focus. Despite observations of differential chemokine expression in colon cancer being associated with poor prognosis, the precise roles of most chemokines in this cancer’s progression are still not fully understood [22,23]. This study utilizes bioinformatics analysis and experimental validation methods to investigate the potential role of chemokines in colon cancer. A prognostic risk model based on chemokines is developed to offer novel insights and approaches for the prognosis and treatment of colon cancer.

## 2. Results

### 2.1. Chemokine Mutation Analysis

**Figure 1:**
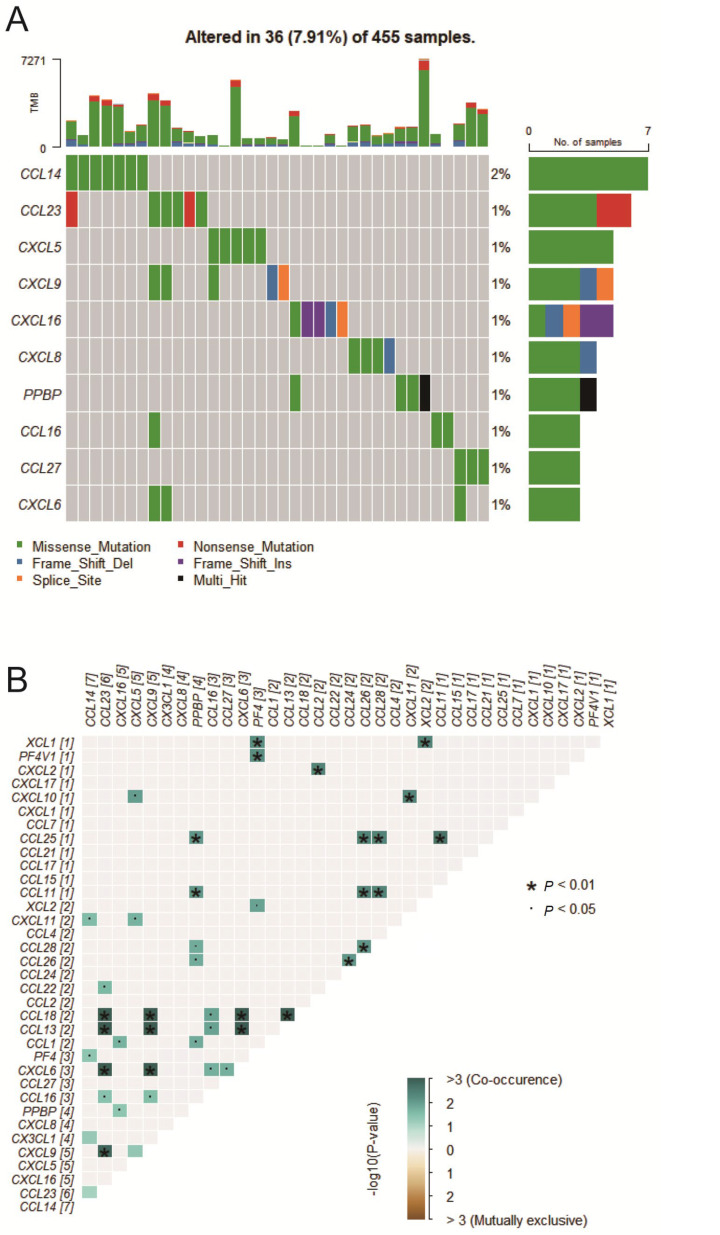
Mutational profiling of chemokines using the TCGA dataset. (**A**) Oncoplot showing the somatic landscape of the top 10 chemokines in the COAD samples from the TCGA database. (**B**) Co-occurring mutation and mutual exclusivity analysis of chemokines.

The analysis of 455 COAD samples identified mutations in chemokines in 36 samples, accounting for 7.91% of the total. Figure 1A illustrates the top ten genes with the highest mutation frequencies, including CCL14, CCL23, CXCL5, CXCL9, CXCL16, CXCL8, PPBP, CCL16, CCL27, and CXCL6. Notably, CCL14 had the highest mutation frequency, exceeding 2%. The analysis found missense, frameshift deletions, and nonsense mutations as the most common types. Moreover, coexisting mutations among chemokines were observed in COAD samples, such as CCL18 and CCL13, CCL13 and CXCL6, as well as CCL18 and CXCL6 (Figure 1B).

### 2.2. Expression of Chemokines in COAD

**Figure 2:**
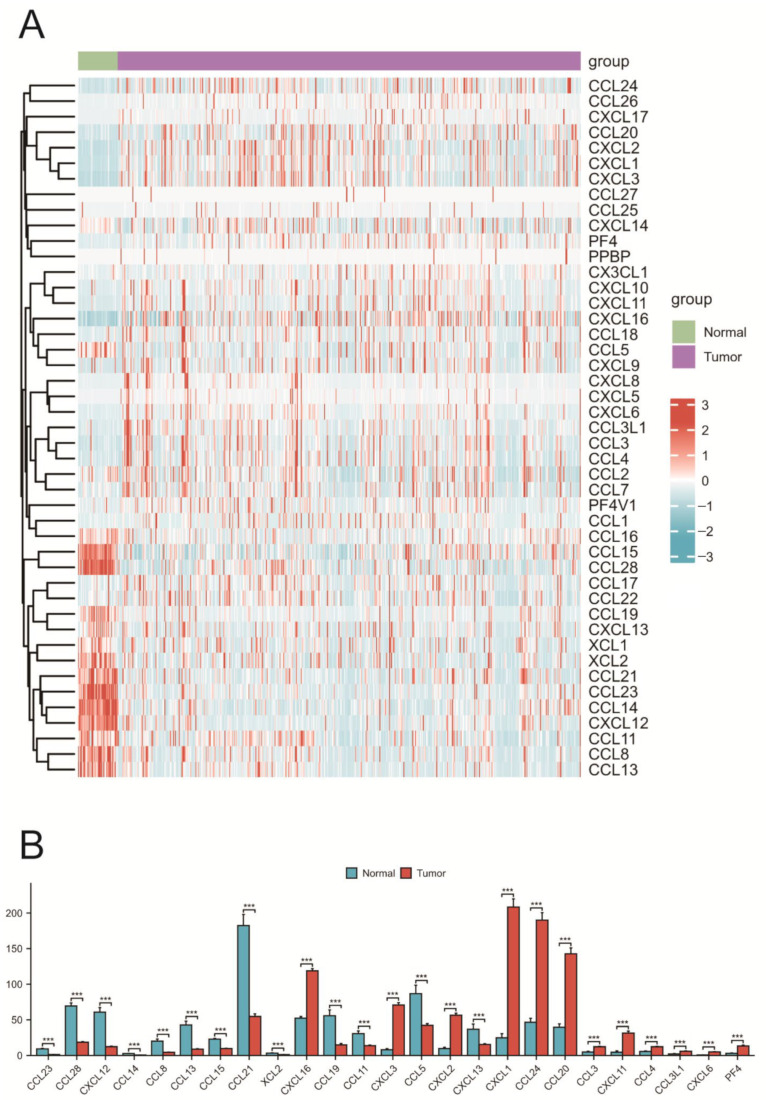
Expression analysis of chemokines. (**A**) Expression levels of chemokines in the TCGA dataset. Red indicates upregulated genes, and blue indicates downregulated genes. (**B**) Differentially expressed chemokines in COAD. *p* < 0.001 (***).

A comparison was made between the expression differences in 46 chemokines in COAD and normal tissues (Figure 2A). The results indicated that 25 chemokines exhibited significant differential expression between COAD and normal tissue samples (Figure 2B). Furthermore, Spearman correlation analysis was used to investigate the correlation among the 46 chemokines in COAD, revealing both positive and negative correlations in their expression. Correlation analysis showed that the strongest positive correlation was between CXCL10 and CXCL9 (R = 0.91), while the strongest negative correlation was between CXCL14 and CCL18 (R = −0.29).

### 2.3. Enrichment Analysis of Chemokines

**Figure 3:**
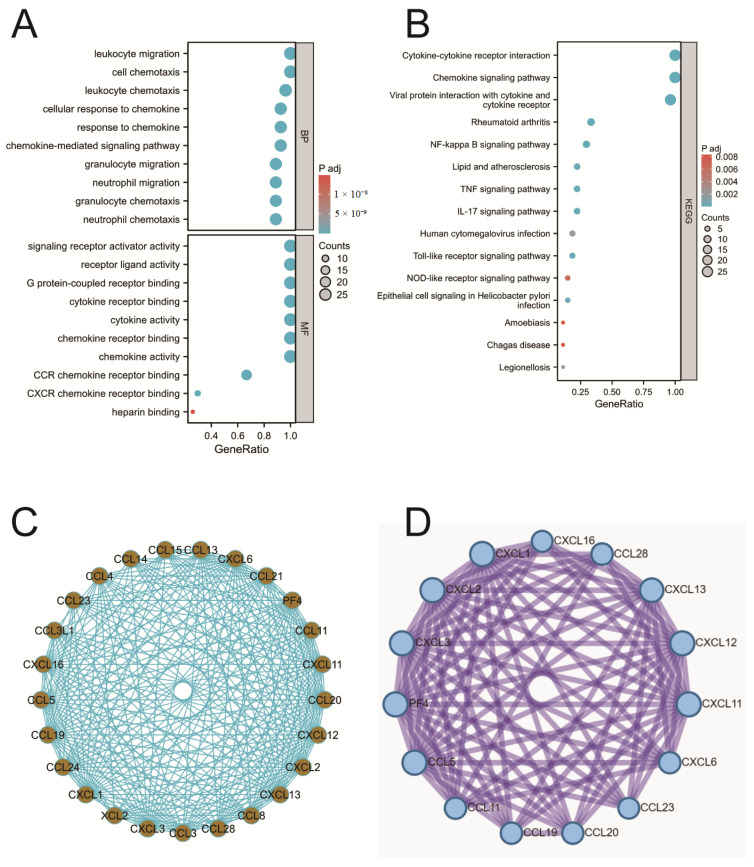
Functional annotation analysis of chemokines. (**A**) GO functional annotation of chemokines. (**B**) KEGG pathway enrichment analysis of chemokines. (**C**) PPI network analysis of chemokines. (**D**) Core gene analysis of chemokines.

To explore the biological functions of chemokines in COAD, GO functional annotation and KEGG pathway enrichment analysis were conducted on differentially expressed chemokines. The results indicated that in COAD, chemokines were involved in several biological processes (BP), including chemokine-mediated signaling pathways, responses to chemokines, cellular responses to chemokines, and neutrophil chemotaxis (Figure 3A). Additionally, chemokines significantly influenced molecular functions (MFs), involving aspects such as chemokine activity, chemokine receptor binding, cytokine activity, and cytokine receptor binding. However, no significant involvement of chemokines was found in the analysis of cellular components (CCs). The KEGG pathway analysis indicated that the functions of chemokines in COAD were closely related to cytokine–cytokine receptor interactions, the NF-κB signaling pathway, and the IL-17 signaling pathway (Figure 3B).

### 2.4. PPI Analysis

In order to investigate the interactions of chemokines in COAD, a protein–protein interaction (PPI) analysis was conducted on differentially expressed chemokines. The results, as depicted in Figure 3C, demonstrated a dense interaction network among these chemokines. By utilizing the MCODE plugin in Cytoscape (v3.10.1) software, 15 core genes were successfully identified within the PPI network, as shown in Figure 3D.

### 2.5. Construction of the Prognostic Risk Model

**Figure 4:**
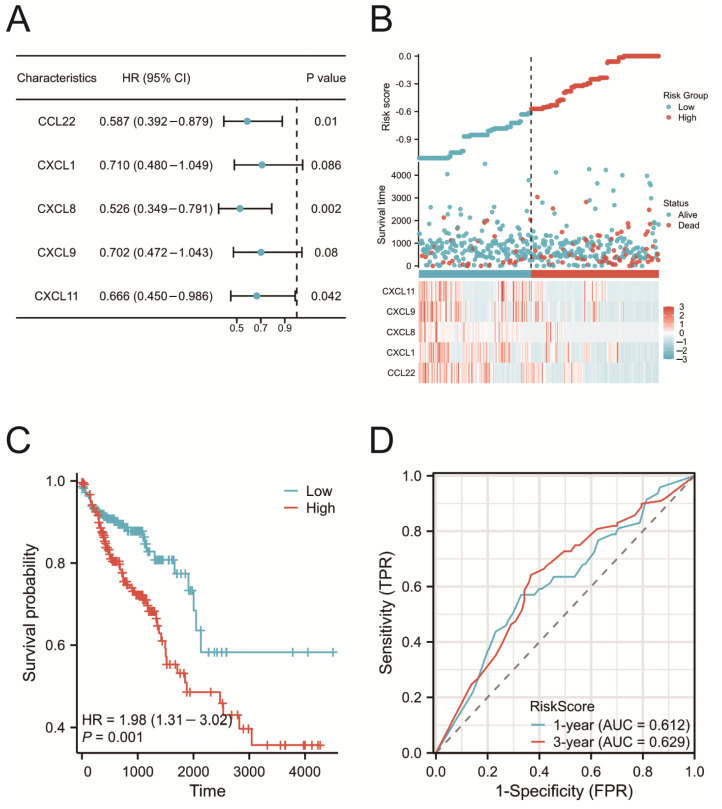
Prognostic value analysis of chemokines. (**A**) Univariate Cox regression analysis between chemokine expression and overall survival. (**B**) Distribution of risk scores based on predictive characteristics. (**C**) Kaplan–Meier survival curves for OS in COAD patients. The red line represents the high-risk group, and the blue line represents the low-risk group. (**D**) Time-dependent ROC curve analysis of chemokines.

Univariate Cox regression analysis was performed on 46 chemokines to evaluate their impact on the prognosis of COAD patients. The analysis results indicated that five chemokines were significantly associated with the survival time of COAD patients (Figure 4A). Furthermore, based on multivariate Cox regression analysis, a prognostic risk model was constructed using five genes (CCL22, CXCL1, CXCL8, CXCL9, and CXCL11), and risk scores were calculated for each patient accordingly. Based on the median risk score, 477 COAD patients were divided into high-risk (238 patients) and low-risk (239 patients) groups. The distribution of risk scores and model gene expression for both groups of patients is presented in Figure 4B. A Kaplan–Meier survival curve analysis revealed that patients in the high-risk group had significantly poorer prognosis (Figure 4C). Additionally, the ROC curve had an AUC of 0.629, indicating that the model had relatively good performance in predicting the prognosis of COAD patients (Figure 4D).

### 2.6. Validation of the Prognostic Risk Model

**Figure 5:**
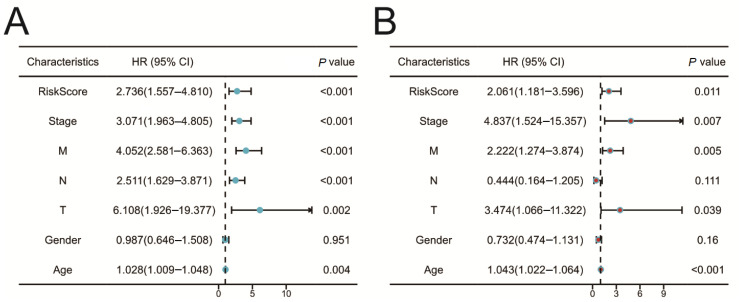
Regression analysis of chemokine prognostic risk models. (**A**) Univariate Cox regression analysis. (**B**) Multiple Cox regression analysis.

Univariate (Figure 5A) and multivariate (Figure 5B) Cox regression analyses were utilized to evaluate the relationship between clinical pathological characteristics and prognosis. The analysis results indicated that the prognostic risk model served as an independent predictive factor in forecasting the prognosis of COAD patients (*p*-value < 0.05).

### 2.7. Protein and mRNA Expression of CCL22, CXCL1, CXCL8, CXCL9, and CXCL11

**Figure 6:**
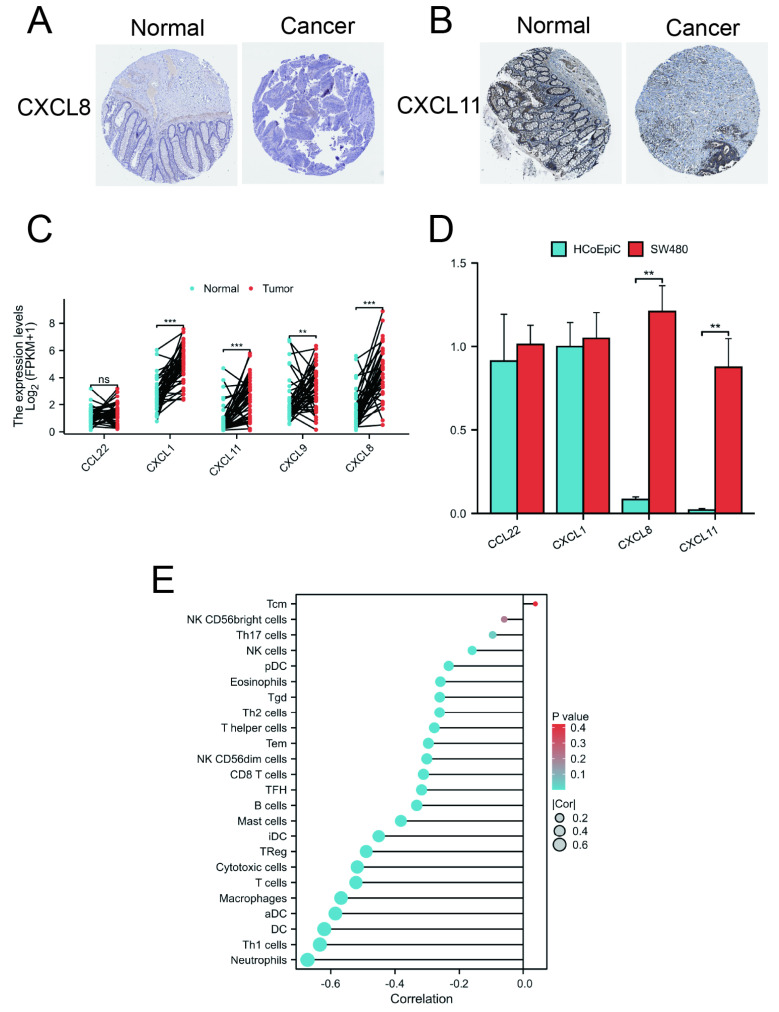
Immunohistochemical and qRT-PCR analysis of core chemokines. (**A**) CXCL8 expression in COAD tissues was higher than normal tissues. (**B**) The expression of CXCL11 in COAD tissues was higher than normal tissues. (**C**) Core chemokine expression in tissues paired in the TCGA COAD dataset. (**D**) qRT-PCR analysis of core chemokines. (**E**) Correlation between risk score and immune cell infiltration. ns (not significant); *p* < 0.01 (**); *p* < 0.01 (***).

To investigate the differences in protein expression of CCL22, CXCL1, CXCL8, CXCL9, and CXCL11 between normal and COAD tissues, protein expression data for these genes were downloaded from the Human Protein Atlas (HPA). The results demonstrated that, in COAD tissues, the protein expression levels of CXCL8 and CXCL11 were significantly higher compared to normal tissues (Figure 6A,B). Due to the absence of protein expression data for CCL22, CXCL1, and CXCL9 in the HPA database, their expression levels are not presented in this paper. Additionally, an analysis of COAD and adjacent non-cancerous tissue samples from the TCGA database confirmed significant increases in CXCL1, CXCL8, CXCL9, and CXCL11 in cancer tissues, while CCL22 showed no significant change (Figure 6C). Furthermore, qRT-PCR experimental results further confirmed that compared with normal colon epithelial cells, the expression levels of CCL22, CXCL1, CXCL8, and CXCL11 were upregulated in colon cancer cells (Figure 6D). It should be noted that CXCL9 was not detected in SW480.

### 2.8. Correlation between Risk Score and Immune Cell Infiltration

As previously mentioned, chemokines play a significant role in inflammatory responses and tumor immunity. To evaluate the correlation between risk score and immune cell infiltration in COAD, ssGSEA analysis was employed. The analysis results indicated significant correlations between the risk score and 22 types of immune cells, except for NK CD56bright and Tcm cells. This finding suggests that the risk score may reflect the cellular composition of the immune microenvironment, closely related to the tumor’s immune response (Figure 6E).

### 2.9. Functional Enrichment Analysis in Different Risk Groups

**Figure 7:**
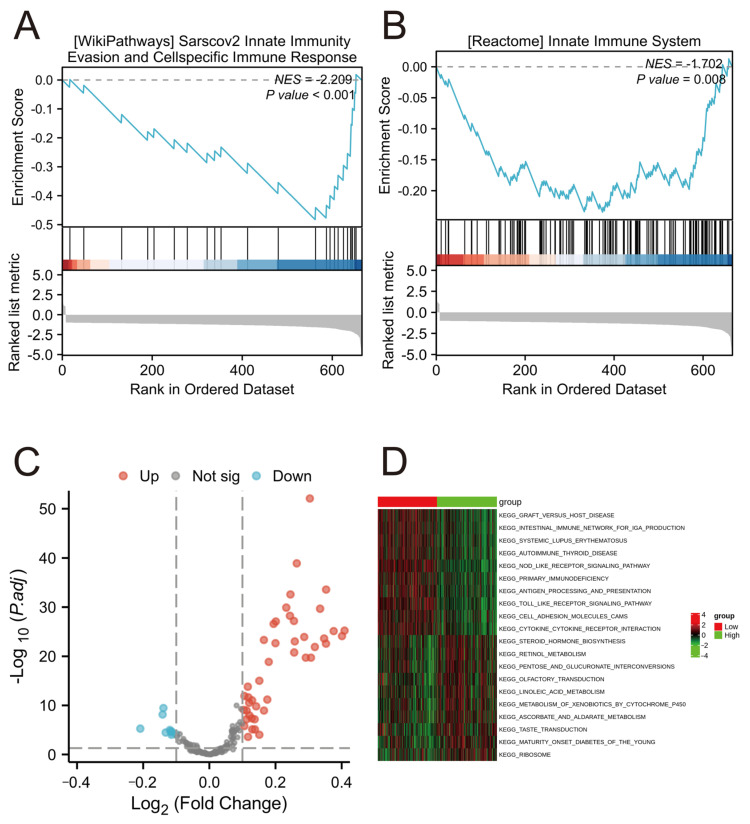
Differential signaling pathway enrichment in different risk groups. (**A**,**B**) Analysis of immune response genomes and immune system processes in high- and low-risk groups by GSEA. (**C**,**D**) GSVA enrichment analysis showing up- and downregulation of signaling pathways in different risk groups. Volcano map (**C**) and heatmap of differential expression in KEGG pathway (**D**). Red color represents upregulated pathways, and green color represents downregulated pathways.

GSEA analysis revealed that, compared to the high-risk group, the low-risk group showed significant enrichment in gene sets “INNATE IMMUNITY EVASION AND CELL-SPECIFIC IMMUNE RESPONSE” and “INNATE IMMUNE SYSTEM” (Figure 7A,B). Additionally, GSVA indicated that several metabolic pathways varied significantly in the high-risk group compared to the low-risk group (Figure 7C,D). These pathways include “KEGG_NOD_LIKE_RECEPTOR_SIGNALING_PATHWAY”, “KEGG_PRIMARY_IMMUNODEFICIENCY”, and “KEGG_ANTIGEN_PROCESSING_AND_PRESENTATION”. The alterations in these pathways may be one of the key factors influencing the prognosis of COAD.

## 3. Discussion

The tumor immune microenvironment plays a crucial role in the anti-tumor activity in COAD. Chemokines, as a significant class of signaling molecules, significantly influence the tumor immune microenvironment by regulating inflammatory responses and immune cell infiltration [8]. COAD is characterized by heterogeneous stromal cells such as endothelial and fibroblast cells, with its microenvironment infiltrated by various immune cells including CD4+ T cells, CD8+ T cells, neutrophils, macrophages, and dendritic cells [24]. These immune cells not only play a role in the production of chemokines but also play a key role in regulating tumor immune responses. During the progression of COAD, studies have shown that the expression levels of certain chemokines, such as CXCL9 and CCL2, are closely associated with the tumor’s invasive behavior and prognosis [25,26]. These chemokines affect tumor growth, metastasis, and immune escape by influencing the recruitment and activation of immune cells. However, current research on the specific roles and mechanisms of most chemokines in the progression of COAD remains limited. Therefore, a comprehensive analysis of chemokines in COAD is crucial.

In this study, we initially analyzed the mutation rates of chemokines using data from the TCGA COAD cohort and found that most chemokines in COAD have low mutation rates. This finding suggests that in COAD, chemokines may regulate tumor development through mechanisms other than genetic mutations. Subsequently, we conducted GO functional annotation and KEGG pathway enrichment analysis on 25 differentially expressed chemokines between normal and COAD tissues to explore their biological functions. GO analysis indicated that these chemokines are involved in several immune-related biological processes, including chemokine-mediated signaling pathways, responses to chemokines, cellular responses to chemokines, and neutrophil chemotaxis. A KEGG pathway analysis further revealed that cytokine–cytokine receptor interactions, the NF-κB signaling pathway, and the IL-17 signaling pathway are closely related to the functions of chemokines in COAD. Notably, recent studies have reported that specific chemokines, such as CXCL8, can enhance the proliferation, migration, and invasion of colon cancer cells by activating the PI3K/Akt/NF-κB signaling pathway, promoting epithelial–mesenchymal transition [27]. This aligns with our findings, further confirming the significant role of chemokines in the pathogenesis of COAD.

Chemokines activate a series of signaling pathways through binding to specific receptors that play a central role in regulating tumor cell proliferation, survival, migration and invasion. Multiple chemokines share key signaling pathways such as NF-κB and PI3K/Akt, which play a role in maintaining the inflammatory microenvironment of tumors and promoting tumor cell survival. For example, both CXCL8 and CCL2 promote the production of inflammatory mediators and the recruitment of immune cells through the activation of the NF-κB pathway, which enhances the inflammatory microenvironment of tumors and the resistance of tumor cells to apoptosis [27,28]. The PI3K/Akt pathway is also co-regulated by several chemokines, including CXCL12, which activates the pathway by binding to CXCR4 and enhances tumor cell resistance to apoptosis by binding to CXCR4 and enhancing the ability of tumor cells to resist apoptotic signals, thereby supporting continued tumor growth and metastasis [29,30,31,32]. However, different chemokines may also act through unique pathways that exhibit specificity in their actions [33]. For example, CCL20, by binding to CCR6, regulates the ability of cells to migrate and invade by activating the RhoA pathway in colorectal cancer, a role not commonly seen in other chemokines [34]. Another example is CXCL12, which, in addition to the PI3K/Akt and JAK/STAT pathways, promotes tumor cell proliferation by activating the Wnt/β-linker protein signaling pathway, which has been associated with tumor invasiveness and treatment resistance [35]. This diversity of signaling mechanisms not only reveals the complex role of chemokines in the development of COAD but also points to a variety of potential signaling pathways that need to be considered when developing targeted therapeutic strategies.

We conducted a PPI network analysis and identified 15 core genes that may play a crucial role in regulating chemokine-related molecular networks in COAD. This finding provides new insights into how chemokines collaboratively function at the molecular level to influence COAD. Through Cox regression analysis, five chemokines (CCL22, CXCL1, CXCL8, CXCL9, and CXCL11) were found to be significantly associated with overall survival (OS) in patients with COAD. A prognostic risk model was developed using these chemokines and validated through Kaplan–Meier survival curves and ROC analysis, demonstrating its effectiveness and accuracy. Our study found that the risk score is an independent predictive factor for the prognosis of COAD.

CCL22 is a key player in cancer-related immune responses, primarily affecting regulatory T cells (Treg) and their interactions with dendritic cells in the tumor microenvironment. Produced mainly by dendritic cells, CCL22 controls the movement of Treg by binding to CCR4 receptors. This mechanism is crucial for modulating T cell immune responses, facilitating the recruitment of Treg to tumor sites and enhancing communication between dendritic cells and Treg in lymph nodes. These interactions contribute to immune suppression, particularly in various cancers [36]. Studies on head and neck squamous cell carcinoma (HNSCC) have identified CCL22 and CCL17 as important prognostic factors for patient survival and response to immune checkpoint blockade therapy. These chemokines are closely linked to the infiltration levels of CD4+ T cells in HNSCC and impact the activation of the mTORC1 signaling pathway in these cells. These findings underscore the significance of the CCL22-CCR4 axis in immune checkpoints and its potential as a target for cancer immunotherapy, especially in treating HNSCC [37]. Additionally, CCL22 plays a vital role in maintaining immune balance, particularly in dendritic cells’ clearance of apoptotic cells. The expression of CCL22 is essential for facilitating contact between dendritic cells and Treg, which is critical for regulating T cell immune responses [37]. Recent research has shown that in colon cancer, CCL2 influences the behavior of immune cells in the tumor microenvironment through its interaction with its primary receptor CCR2. Activation of the CCL2-CCR2 axis results in the migration of immune cells, notably, macrophages and myeloid-derived suppressor cells (MDSCs), into the tumor microenvironment [26].

CXCL1, a member of the CXCL chemokine family, is involved in various stages of tumor progression, such as the regulation of immune cell activity, tumor cell proliferation, invasion, metastasis, and formation of tumor-associated microvasculature [38]. In various cancers, CXCL1 displays distinct functions. For example, in lung cancer, it works in conjunction with other CXCL family members to enhance the recruitment of tumor-associated neutrophils, impacting tumor cell growth and angiogenesis, which are critical for lung cancer development. Overexpression of CXCL1 in gastric cancer is associated with increased migration and invasion of gastric cancer cells, indicating that CXCL1 and its receptors could be potential targets for gastric cancer therapy [39]. In breast cancer, CXCL1 originating from tumor-associated macrophages (TAMs) promotes tumor metastasis by activating signaling pathways such as NF-κB/SOX4. Its ability to promote metastasis is not solely dependent on the CXCR2 receptor, highlighting the multifaceted and complex nature of CXCL1 in cancer progression [40]. Recent research has also revealed the significant involvement of CXCL1 in colon cancer progression, where it facilitates cancer development through the activation of the NF-κB/P300 signaling pathway [41].

CXCL8, a chemokine, plays a crucial role in cancer biology by regulating immune responses within the tumor microenvironment. Recent studies have revealed the role of CXCL8 in the progression of triple-negative breast cancer (TNBC). CXCL8 modulates the TNBC immune microenvironment by controlling the infiltration of CD4+ and CD8+ T cells and increasing CD274 (PD-L1) expression, unveiling potential therapeutic targets for anti-tumor immunotherapy [42]. The function of CXCL8 is closely tied to its interaction with the CXCR1 and CXCR2 receptors. While these receptors share high sequence homology, they exhibit distinct roles upon CXCL8 binding. This interaction triggers conformational changes in the receptors, initiating essential signaling cascades crucial for tumor motility, angiogenesis, and survival. Notably, CXCL8 activation of the PI3K/Akt signaling pathway is a critical process in various cancers, such as androgen-independent prostate cancer [43]. In colon cancer, CXCL8 is implicated in cellular proliferation, invasion, and metastasis. By activating the PI3K/Akt/NF-κB signaling pathway, CXCL8 can induce epithelial–mesenchymal transition, promoting colon cancer progression [27]. The CXCL8-CXCR2 axis in the colon cancer microenvironment is also significant, with studies demonstrating that elevated CXCL8 levels in serum and the tumor microenvironment enhance human and murine colon cancer cell growth and support pulmonary and hepatic metastasis [44].

The functions of CXCL9 are multifaceted, encompassing not only the behaviors of immune cells but also their interactions with tumor cells. Taking prostate cancer as an example, CXCL9 promotes tumor progression by inhibiting cytokines produced by T cells, which is evident in the severity of pathology, increased cellular proliferation, and reduced patient survival rates. Additionally, studies have found a positive correlation between the expression of CXCL9 and the clinical pathological stages of prostate cancer [45]. Further exploration of the role of CXCL9 in the tumor microenvironment reveals its involvement in various processes such as angiogenesis, metastasis, and inflammatory response. Through its interaction with the receptor CXCR3, CXCL9 affects the behavior of various immune cells within the tumor, particularly T cells and NK cells [46]. This chemokine plays a key role in regulating the migration and activity of these immune cells, thereby impacting the overall immune response to cancer. The involvement of CXCL9 in various stages of tumor development and its impact on different cell types in the tumor microenvironment highlight its functional complexity. Recent studies indicate that in colon cancer tissues, CXCL9 expression levels are significantly higher than in normal colonic tissues and are notably associated with tumor differentiation, invasion, lymph node metastasis, distant metastasis, and vascular invasion [25]. Importantly, high CXCL9 expression correlates positively with colon cancer patient survival rates, suggesting its potential as an independent prognostic factor.

CXCL11 plays a significant role in modulating the tumor microenvironment and influencing the efficacy of cancer immunotherapy. In the tumor microenvironment, CXCL11 is renowned for its multifunctionality, including inhibiting angiogenesis, affecting the proliferation of various cell types, and promoting the migration of certain immune cells. Moreover, CXCL11 contributes to the promotion of cancer invasion driven by fibroblasts through enhancing cell adhesion and suppressing M2 macrophage polarization, thereby influencing tumor progression [47]. In a comprehensive analysis of various cancer types, it was found that the expression of CXCL11 in tumor tissues is significantly higher than in normal tissues in most cancers. This increased level of expression is associated with different prognostic outcomes in various cancers. Notably, CXCL11 is positively associated with the infiltration of CD8+ T cells and follicular helper T cells in the tumor microenvironment, indicating its involvement in immune cell infiltration and potential impact on immunotherapy efficacy [48]. In the context of colon cancer, high CXCL11 expression is linked to increased levels of anti-tumor immune cells and particularly activated CD8+ T cells and natural killer cells while decreasing the proportion of tumor-promoting immune cells, suggesting a favorable role of CXCL11 in the anti-tumor immune response [49]. Additionally, in colon cancer cells, the overexpression of RBP-Jκ leads to the secretion of CXCL11, which, in turn, promotes cancer cell migration induced by tumor-associated macrophages (TAMs) through TGF-β1 secretion. This highlights the significance of the interplay between RBP-Jκ and CXCL11 in the context of colon cancer cells and TAMs [50].

GSEA results indicated that in COAD, the low-risk group displayed significant enrichment in gene sets associated with “INNATE IMMUNITY EVASION AND CELL-SPECIFIC IMMUNE RESPONSE” and “INNATE IMMUNE SYSTEM” compared to the high-risk group. This observation suggests that patients in the low-risk category may possess a more robust innate immune response, potentially aiding in combating cancer development and progression. Activation of the innate immune system is essential for enhancing immune cell capabilities in identifying and eliminating cancer cells, which could play a crucial role in slowing tumor advancement. On the other hand, GSVA revealed significant differences in the activity of multiple metabolic pathways between the high-risk and low-risk groups. These pathways include “KEGG_NOD_LIKE_RECEPTOR_SIGNALING_PATHWAY”, “KEGG_PRIMARY_IMMUNODEFICIENCY”, and “KEGG_ANTIGEN_PROCESSING_AND_PRESENTATION”, among others. In the high-risk group, these pathways exhibited upregulation or downregulation, suggesting that alterations in these pathways could be key factors influencing COAD prognosis. For example, the upregulation of the NOD-like receptor signaling pathway may promote inflammatory responses and alterations in the tumor microenvironment, while adjustments in the antigen processing and presentation pathway could affect the efficiency of the immune system in recognizing and eliminating tumor cells [51,52].

Although this study has made some advancements in exploring the role of chemokines in COAD, its methodology has some non-negligible limitations. Firstly, as a retrospective study, its results are constrained by the availability and quality of historical data. To more comprehensively validate these findings, large-scale prospective studies are required. These studies could provide more real-time data on the role of chemokines in COAD development, improving the predictive capacity of prognostic risk models and the effectiveness of clinical applications. Additionally, further experimental validation is needed to determine the specific biological functions and mechanisms of action of these five chemokines in COAD. Although previous studies have indicated the importance of chemokines in tumor biology, their mechanisms of action in the specific context of COAD are not yet fully understood.

## 4. Materials and Methods

### 4.1. Data Download and Processing

TCGAbiolinks is an R package that simplifies the access, preparation, and analysis of he Cancer Genome Atlas (TCGA) data. It facilitates the direct download and preprocessing of various data types from the TCGA database, such as gene expression, mutations, copy number variations, and epigenetic data [53]. On the other hand, Maftools is another R package that focuses on the analysis, interpretation, and visualization of Mutation Annotation Format (MAF) files within the context of cancer genomics [54]. This toolset includes features like mutation load calculation, mutational spectrum analysis, and identification of mutant genes. Additionally, Maftools offers diverse data visualization capabilities, including oncoplots, which aid in the identification of significant genetic variations and potential therapeutic targets in cancer research. In this study, the TCGAbiolinks package was utilized to retrieve variant data for COAD from the TCGA database, while the Maftools package was employed for detailed visual analysis and interpretation of the acquired data.

### 4.2. Expression of Chemokines in COAD

Linear Models for Microarray Data (Limma) is a widely used R package designed for differential expression analysis of microarray and high-throughput genomic data [55]. It utilizes linear models and Bayesian methods to effectively handle datasets with small sample sizes, while also supporting complex experimental designs. Limma is a widely used tool for analyzing high-throughput technologies, including RNA-Seq, and offers a variety of statistical methods and data visualization tools. Additionally, the pheatmap package is available for generating customizable heatmaps to visualize complex bioinformatics data. The pheatmap package supports clustering analysis, color grading, and data normalization, allowing biologists and data analysts to present and interpret gene expression patterns and other multi-dimensional datasets intuitively. In this study, we downloaded COAD RNAseq sequencing data from the TCGA database using the TCGAbiolinks package. Subsequently, we employed the Limma package to identify differentially expressed chemokines based on the criteria of adjusted *p*-value < 0.05 and |log2FC| > 1. Utilizing the pheatmap package, we created heatmaps to visually depict the expression levels of these chemokines.

### 4.3. Enrichment Analysis of Chemokines

ClusterProfiler is an R package designed for Gene Set Enrichment Analysis in bioinformatics [56]. It provides tools for comparing and visualizing enrichment results of various biological terms and pathways, supporting multiple public databases such as KEGG, GO, and Reactome. ClusterProfiler can assist researchers in identifying genes and pathways associated with specific biological processes, molecular functions, or cellular components by processing data from gene expression studies. The ClusterProfiler package was used to analyze differentially expressed chemokines for GO functional annotations and KEGG pathway enrichment analysis to explore their biological functions in COAD.

### 4.4. Protein–Protein Interaction Analysis of Chemokines

STRING is a bioinformatics resource that compiles and integrates protein–protein interaction data [57]. It provides a vast array of information from experiments, computational predictions, and public literature, making it a valuable tool for studying protein functions and networks. The STRING database supports interaction network analysis within the genomic scope and provides tools to explore functional associations between proteins, aiding in the understanding of complex molecular mechanisms within cells. In this study, the STRING database was utilized to analyze the protein–protein interactions of differentially expressed chemokines.

### 4.5. Construction of the Prognostic Risk Model

Univariate Cox regression analysis was used to identify chemokines with prognostic value. Subsequently, a prognostic risk model was developed based on these chemokines using multivariate Cox regression analysis, and individual risk scores were calculated for each patient. The formula for the prognostic risk model is as follows: Risk score = CCL22 × (−0.321) + CXCL1 × (−0.059) + CXCL8 × (−0.473) + CXCL9 × (−0.016) + CXCL11 × (−0.234). Patients were stratified into high-risk and low-risk groups according to the median of their risk scores. Kaplan–Meier survival curves were employed to evaluate the disparities in overall survival (OS) between these two groups. Moreover, the accuracy of the prognostic risk model was confirmed through the use of ROC curves.

### 4.6. Immunohistochemical Analysis

The Human Protein Atlas (HPA) is a comprehensive scientific project aimed at systematically mapping the distribution and expression of all human proteins in tissues, cells, and diseases [58]. By integrating high-throughput proteomics, imaging technologies, and bioinformatics analysis, HPA provides invaluable resources and insights for the study of human biology and diseases. Immunohistochemical information of chemokines in the prognostic risk model was retrieved using the HPA database.

### 4.7. Cell Culture

The human colon cancer cell line SW480 and the normal human colonic epithelial cell line HCoEpiC were provided by the American Type Culture Collection (Manassas, VA, USA). These cell lines were cultured in Dulbecco’s Modified Eagle’s Medium (DMEM; Gibco, Invitrogen Life Technologies, Carlsbad, CA, USA) supplemented with 10% fetal bovine serum (FBS). The cells were maintained in an environment of 37 °C and 5% CO_2_, with passaging every 2 to 3 days.

### 4.8. Quantitative Real-Time PCR (qRT-PCR)

Total RNA was extracted from the samples using Trizol reagent (Invitrogen). Subsequently, RNA concentration and purity were measured using a Nanodrop spectrophotometer. cDNA synthesis was carried out with a one-step RT kit containing gDNA Eraser (Takara, Osaka, Japan). The quantitative real-time PCR reactions were performed using TB Green Premix Ex TaqII (Takara, Japan) on a 7900 Fast Real-Time PCR System (Applied Biosystems, Waltham, MA, USA). The expression levels of target genes were calculated using the 2^−ΔΔCt^ method. Primer sequences used in the experiments were as follows: CCL22 (forward TCCTGGGTTCAAGCGATTCTCC, reverse GTCAGGAGTTCAAGACCAGCCT), CXCL1 (forward AGCTTGCCTCAATCCTGCATCC, reverse TCCTTCAGGAACAGCCACCAGT), CXCL8 (forward GAGAGTGATTGAGAGTGGACCAC, reverse CACAACCCTCTGCACCCAGTTT), CXCL9 (forward CTGTTCCTGCATCAGCACCAAC, reverse TGAACTCCATTCTTCAGTGTAGCA), CXCL11 (forward GCCTCCATAATGTACCCAAG, reverse GCCTTGCTTGCTTCGATTTG), and GAPDH (forward GTCTCCTCTGACTTCAACAGCG, reverse ACCACCCTGTTGCTGTAGCCAA).

### 4.9. Correlation between Risk Score and Immune Cell Infiltration

Single Sample Gene Set Enrichment Analysis (ssGSEA) is an analytical tool used to quantify the expression activity of predefined gene sets in individual samples. This method is widely used to study individualized gene expression variations, aiming to explore molecular mechanisms under different biological states or disease conditions [59]. ssGSEA was utilized to assess the level of immune cell infiltration in COAD samples and to explore the correlation between risk score and immune cell infiltration.

### 4.10. GSEA and GSVA Functional Annotation

Gene Set Enrichment Analysis (GSEA) is implemented in R using the GSEA package, providing a computational framework for deciphering biological signals in gene expression data [60]. This method identifies gene sets significantly enriched under specific biological conditions, revealing potential biological pathways and functional modules. Additionally, Gene Set Variation Analysis (GSVA) is a non-parametric, unsupervised gene set enrichment method, also implemented in the R environment [61]. GSVA enables the assessment of gene set expression level variations at the level of individual samples, thus exploring differences in biological processes, pathways, and functional clusters under various conditions or phenotypes. The chemokines were analyzed using the GSEA and GSVA packages.

### 4.11. Statistical Analysis

All statistical analyses were conducted using the R (v4.2.2) software [62]. *p*-value < 0.05 was considered statistically significant.

## 5. Conclusions

This study, through comprehensive analysis, has successfully revealed the significant biological functions and prognostic value of chemokines in COAD, subsequently leading to the construction of a prognostic risk model based on these factors. The risk scoring of the model effectively reflects the immune microenvironment status of COAD patients, particularly in aspects of immune response and KEGG signaling pathways. Our findings not only enhance the understanding of the complexity of the COAD immune microenvironment but also offer potential prognostic and therapeutic biomarkers, providing new insights for the development of personalized treatment strategies.

## Data Availability

All datasets used in this study are publicly available on the TCGA.
